# Pediatric Early Warning Systems (PEWS) improve provider‐family communication from the provider perspective in pediatric cancer patients experiencing clinical deterioration

**DOI:** 10.1002/cam4.5210

**Published:** 2022-09-21

**Authors:** Srinithya R. Gillipelli, Erica C. Kaye, Marcela Garza, Gia Ferrara, Mario Rodriguez, Dora Judith Soberanis Vasquez, Alejandra Mendez Aceituno, Federico Antillón‐Klussmann, Jami S. Gattuso, Belinda N. Mandrell, Justin N. Baker, Carlos Rodriguez‐Galindo, Asya Agulnik, Dylan E. Graetz

**Affiliations:** ^1^ Baylor College of Medicine Houston Texas USA; ^2^ Department of Global Pediatric Medicine St. Jude Children's Research Hospital Memphis Tennessee USA; ^3^ Division of Quality of Life and Palliative Care St. Jude Children's Research Hospital Memphis Tennessee USA; ^4^ Department of Oncology Unidad Nacional de Oncología Pediátrica Guatemala City Guatemala; ^5^ Department of Nursing Unidad Nacional de Oncología Pediátrica Guatemala City Guatemala; ^6^ Department of Critical Care Unidad Nacional de Oncología Pediátrica Guatemala City Guatemala; ^7^ School of Medicine Francisco Marroquin University Guatemala City Guatemala; ^8^ Department of Nursing Research St. Jude Children's Research Hospital Memphis Tennessee USA

**Keywords:** cancer management, clinical cancer research, clinical guidelines, medical oncology, pediatric cancer

## Abstract

**Background:**

Communication between providers and patients' families is an integral part of clinical care. Family concern is a validated component of Pediatric Early Warning Systems (PEWS); however, little is known about the impact of PEWS on provider‐family communication.

**Methods:**

Semi‐structured interviews were conducted with 83 ward and Pediatric Intensive Care Unit (PICU) providers involved in the care of patients with deterioration at two pediatric oncology hospitals of different resource levels: St. Jude Children's Research Hospital (*n* = 42) in the United States and Unidad Nacional de Oncología Pediátrica (UNOP, *n* = 41) in Guatemala. Interviews were conducted in the participants' native language (English or Spanish), transcribed, and translated into English. Transcripts were coded by two researchers and analyzed for thematic content surrounding family communication and concern.

**Results:**

All participants recognized patients' families as a valuable part of the care team, particularly during events requiring escalation of care. Perceived barriers to communication included limited time spent at the bedside, and, at UNOP, language and literacy challenges which occasionally limited providers' ability to assess family concern and involve families in patient care. Despite these barriers, providers perceived PEWS improved communication by facilitating more interaction with families, allowing for relationship‐building, anticipatory guidance, and destigmatization of the PICU. PEWS assessments also allowed families to contribute to identification of deterioration.

**Conclusions:**

PEWS improve the quality of communication between providers and families by providing more opportunities for interaction, building relationships, and trust. These findings further support the use of PEWS in the care of children with cancer in hospitals of all resource‐levels.

## Graphical abstract

In this qualitative analysis of provider interviews, we identify PEWS as facilitators of communication between providers and patient families in resource‐limited and high‐resource pediatric oncology hospitals. Our findings demonstrate that providers perceive the family concern component of PEWS allows greater inclusion of patient families as a part of the healthcare team.

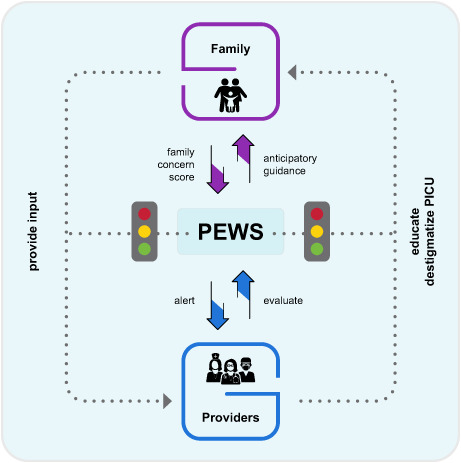

## 1 INTRODUCTION

High‐quality communication between providers and patient families is an integral part of family‐centered pediatric care,^1^ particularly during clinical deterioration.^2^ There are, however, major barriers^3^, ^4^, ^5^ to family‐centered communication, including family beliefs, health literacy, and provider‐family hierarchy.^6^ In high‐income settings, these communication barriers are primarily related to institutional factors.^7^ There is limited literature describing barriers to family‐centered communication in low‐ and middle‐income countries.

Pediatric Early Warning Systems (PEWS) are bedside assessment tools that allow for early identification of clinical deterioration using a scoring tool and action algorithm.^8^ PEWS decrease clinical deterioration events and Pediatric Intensive Care Unit (PICU) utilization^9^ in both high‐resource and resource‐limited settings.^10^ They also improve providers' perceived quality of care,^11^ provider emotions around deterioration,^12^ and interdisciplinary communication.^10^

The influence of PEWS on family‐centered communication, however, remains unknown. Understanding how PEWS impact inclusion of families will clarify the impact of PEWS on family‐centered communication and improve patient care. In this study, we examine the relationship between PEWS and provider‐family communication during clinical deterioration at two pediatric oncology centers of different resource levels, one in the United States and one in Guatemala.

## 2 METHODS

### 2.1 Setting

Details about the setting, population, and data collection of this study have previously been described.^10^, ^11^, ^12^ The objective of the primary study was to understand the effect of PEWS on care communication between multidisciplinary clinician members of the care team in settings of different resource levels.^10^ Another study investigated the effect of PEWS on clinicians' emotional responses to patient deterioration events.^12^ Most recently, these data were analyzed to understand the impact of PEWS on the quality of care during deterioration events.^11^ In this study, we particularly focus on the impact of PEWS on family‐centered care and communication between providers and patient families during pediatric cancer clinical deterioration events in high‐resource and resource‐limited settings. Providers who regularly care for hospitalized pediatric oncology patients were interviewed at two pediatric oncology centers: St. Jude Children's Research Hospital (St. Jude) and Unidad Nacional de Oncología Pediátrica (UNOP).

St. Jude is in Memphis, TN, USA and is considered a high‐resource setting. The patients at St. Jude are primarily from the United States: 21.4% from Shelby County in Memphis; 30.8% from the surrounding Memphis area; and 59.9% from the rest of the United States.^13^ Over 90% of children in the surrounding Memphis area are insured, but only about 50% receive ongoing, coordinated, comprehensive care within a medical home.^13^ St. Jude has professional interpreter resources available for seven languages in‐person, 41 languages by video, and 100 languages by audio when necessary.

UNOP is located in Guatemala City, Guatemala, an upper middle‐income country. Due to insufficient staffing and infrastructure, UNOP is considered a low‐resource setting. Nearly 75% of UNOP families are in the lowest three categories of socioeconomic status classification based on income, housing, water supply, and sanitation.^14^ The primary language spoken in Guatemala City is Spanish, but the patients at UNOP speak 20 regional dialects. Approximately 72.8% of UNOP patients are Ladino and speak Spanish, 27.1% are Indigenous and speak dialects other than Spanish, and 0.1% are Caucasian.^15^ Several providers speak a Mayan dialect in addition to Spanish, but professional interpreters for Mayan dialects are not available; informal interpreters including providers and patient family members are utilized when possible.

Despite different resource‐levels, these hospitals share similar missions, patient populations, patient volume, and underwent a similar process of PEWS implementation. PEWS were implemented at UNOP in 2014 as “EVAT” (Escala de Valoración de Alerta Temprana)^16^ and as “sJAWS” (St. Jude Advanced Warning System) at St. Jude in 2016.^17^ The PEWS scoring tool and algorithm are included as Figures S1, S2. Patients are regularly assessed using this tool and scored on five components^16^: Behavior/neurologic, cardiovascular, respiratory, staff concern, and family concern. ‘Family concern’ is documented by the bedside nurse based on a discussion with the patient's caregiver, and the presence of family concern has been validated to predict need for unplanned PICU transfer in both high‐resource and resource‐limited settings.^9^, ^16^, ^18^ PEWS results over five trigger a Rapid Response Team (including PICU consult) at St. Jude, and a PICU consult at UNOP.

**FIGURE 1:**
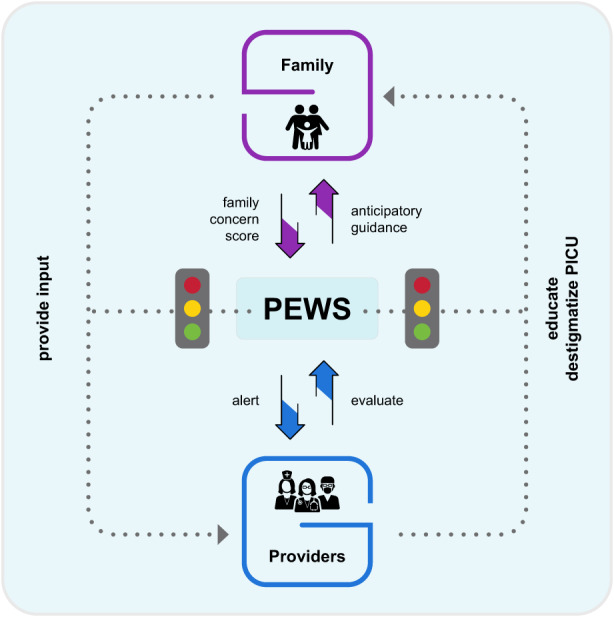
Pediatric early warning systems (PEWS) facilitate communication between providers and families. As families contributed to the family concern score in PEWS to alert providers, providers evaluated the patient to contribute to PEWS, allowing them to provide anticipatory guidance to families. Through this process PEWS facilitated the communication which allowed families to provide input to providers, and providers to educate families and destigmatize the Pediatric Intensive Care Unit (PICU).

### 2.2 Study participants

Study participants included multidisciplinary staff recently involved in a patient deterioration event (an unplanned transfer to the PICU), including bedside nurses and unit nursing coordinators, frontline physicians (pediatricians and pediatric hematology‐oncology fellows), and critical care providers (attending physicians and fellows). Critical care participants from St. Jude also included advanced practice practitioners and critical care nursing coordinators. Provider‐family communication was analyzed from the clinician perspective.

### 2.3 Data collection

Study personnel not involved in PEWS implementation or the discussed deterioration events recruited potential participants. Semi‐structured interviews (see Figure S3 for Interview Guide) were conducted between October and December 2018 in the participants' native language (Spanish or English). The study team included Guatemalan investigators who understand the cultural context and work at UNOP. The team collaborated with psychologists and social workers in Guatemala who are involved with indigenous populations. Interviews were simultaneously transcribed and translated into English using a professional service.

### 2.4 Analysis

Transcribed interviews underwent analysis utilizing inductively derived themes^19^ (see Table S1 for relevant code definitions). Segments coded as “family communication” and “parent concern” were explored. “Family communication” described communication directed to or received from the patient and family regarding the patient's care, and “parent concern” referenced the point given in the PEWS score as described above. Content analysis was conducted to identify and compare themes across disciplines (nursing, ward physicians, PICU providers) and hospital sites (UNOP and St. Jude). MAXQDA software (VERBI GMBH) was used for data management. Consolidated criteria for reporting qualitative research guidelines^20^ were followed to ensure quality in qualitative reporting.

### 2.5 Human subjects

This study was reviewed and deemed exempt from full review by the St. Jude Institutional Review Board and approved by UNOP Human Subjects Oversight Committee. Verbal consent was obtained from participants in English or Spanish. No identifying information was collected from participants and transcripts were de‐identified prior to analysis.

## 3 RESULTS

We conducted 83 interviews: 42 at St. Jude and 41 at UNOP. Table 1 describes participant demographics at both institutions. Analysis revealed three main themes of provider perceptions related to family‐centered communication, including “role of family in care of patients with deterioration,” “barriers to communication with families around deterioration,” and “impact of PEWS on family communication.”

**TABLE 1:** Demographics of interviewed participants

Health care provider	St. Jude *n* (%)	UNOP *n* (%)
Nurses	13 (31)	20 (49)
Coordinator	2 (5)	8 (20)
Bedside nurse	11 (26)	12 (29)
Ward physicians	16 (38)	14 (34)
Oncology fellow	6 (14)	6 (14)
Resident/pediatrician	3 (7)	8 (20)
APP (NP, PA)	7 (17)	N/A
PICU provider	13 (31)	7 (17)
PICU nurse	2 (5)	N/A
APP (NP, PA)	5 (12)	N/A
PICU fellow	N/A	6 (15)
PICU attending physician	6 (14)	1 (2)
Total	42 (100)	41 (100)

*Note*: Adapted from Graetz et al 2021, *“Provider Emotions Surrounding Pediatric Oncology Patient Deterioration”*.

Abbreviations: APP, advanced practice provider; N/A, not available; NP, nurse practitioner; PA, physician assistant; PICU, pediatric intensive care unit; St. Jude, St. Jude Children's Research Hospital; UNOP, Unidad Nacional de Oncología Pediátrica.

The complex relationship between PEWS, families, and providers is depicted in Figure 1.

### 3.1 Role of family in care of patients with deterioration

Providers at both institutions and across disciplines valued family input in the care of deteriorating children. According to providers, parents knew their child better than anyone else, and were the first to notice when their child deviated from a “normal state”: *“their relatives…they tell us if [they're] worried or not for their patients, so it's…easier to detect if they have noticed if there is something different with their children”* (ward physician, UNOP).

In‐depth communication and the inclusion of families as part of the care team enabled relationship‐building and increased family comfort: *“[PICU providers] explained to dad why we were going upstairs, what we wanted to monitor, that we were just being extra cautious. That made them feel comfortable, so it went really smoothly”* (nurse, St. Jude). Involving families also led to personal discussions, forming connections and engendering trust; *“I remember him also because his mom talked a lot about him studying in military school. Then she always told me to take care of her soldier”* (PICU provider, UNOP). Table 2 includes additional quotations representing perceptions of providers regarding the role of the family in the care of patients with deterioration.

**TABLE 2:** Role of family in care of patients with deterioration

Importance of family input in identifying deterioration	St. Jude	*“I always think it's beneficial and super important that you listen to parents, they know their children better than anybody” (ward provider, St. Jude)*
		*“I think the most important thing [nurses] can do on their job is listen to the parents: ‘this has changed, this is concerning’” (ward provider, St. Jude)*
	UNOP	*“There are people who have more feeling with the family, there are other people who speak only when it is necessary, but anyway, we all talk to the relatives. I think [speaking with the family member is] an area where no one can be negative or stay away” (PICU provider, UNOP)*
		*“We even ask parents…how was the patient…and then we can more or less identify how the patients are going to react…” (nurse, UNOP)*
Inclusion of family in care team	St. Jude	*“And again, it gets everybody in one room, so you are all looking at the patient with the parents, with the nurses, with the doctor, with the PICU and communicating about what's going on when numbers are higher” (ward provider, St. Jude)* *“I grabbed Mom and Dad to come out, but the team that was originally out there was just trying to figure out their plan first and then we grabbed them to be a part of the conversation” (nurse, St. Jude)*
	UNOP	*“We evaluate [the patient], we check*…*everything*…*what was [their] [PEWS] scale*…*we always talk to [their] parents to see what they have been noticing in their child and yes is a communication with everybody. (PICU provider, UNOP)*

Abbreviations: PEWS, pediatric early warning systems; PICU, pediatric intensive care unit; St. Jude, St. Jude children's research hospital; UNOP, Unidad Nacional de Oncología Pediátrica.

### 3.2 Barriers to communication with families around deterioration

Providers at both institutions identified provider‐level (perceived by providers to originate in themselves or the institution) and family‐level barriers (perceived by providers to be originating in the family) (Table 3).

**TABLE 3:** Barriers to provider‐family communication

Provider‐level barriers	Time spent educating families	*“[the night nurse and I explained], ‘okay this is the time we are going to send you and this is where you are going, and this is why you are going,’” (nurse, St. Jude)*.
		*“Sometimes the PICU doctor will explain [PICU transfer]…but they are also not the greatest at explaining things in an understandable manner” (nurse, St. Jude)*.
		*“I was the first eight hours of the day, so I think that just having that communication with Mom and being there and watching him and seeing him you know, have bright spots throughout the day” (nurse, St. Jude)*
Family‐level barriers	Language	*“there are linguistic barriers, that makes communication very difficult in that aspect” (nurse, UNOP)* *“There are relatives who have a language barrier, so [communication is] more difficult” (nurse, UNOP)* *“Sometimes, language is a limitation of communication with parents” (nurse, UNOP)* *“When, of course it's a little bit hard…[because of] the little understanding of what cancer is, and [because of] the linguistic barrier, it's a little hard” (nurse, UNOP)*
	Education and culture	*“most [families] understand and are worried, but if I tell them that the child has [a PEWS score] of 6 they will not understand, some of the family members do not speak Spanish or because their cultural level” (ward physician, UNOP)* *“It happens that our families…are of low education, so they do not understand many things” (nurse, UNOP)* *“It's about trying to make the explanations as easy as possible, so they understand, almost with little pictures and things like that” (nurse, UNOP)*

Abbreviations: PEWS, pediatric early warning systems; PICU, pediatric intensive care unit; St. Jude, St. Jude children's research hospital; UNOP, Unidad Nacional de Oncología Pediátrica.

#### 3.2.1 Provider‐level barriers

At both institutions, providers perceived that the amount of time spent interacting with patients and their families affected the quality of provider‐family communication. Shorter interactions were a barrier to high‐quality communication, while providers with more family interaction had enhanced communication. More time spent with the family facilitated relationship‐building and provided an opportunity to explain complex subjects such as PICU procedures: *“sometimes the nurses at direct care have a little more relationship and because they are at intermediate care, [families] spend a lot of time and get to know all the nurses”* (PICU provider, UNOP).

Providers from different disciplines reported spending varying amounts of time with patients. At both institutions, providers perceived nurses spending the most time communicating with families and PICU providers spending the least. A PICU provider at UNOP stated, *“In intensive [care], [communication] is poor because they are regularly addressing the patient, making interventions and the communication we have with parents at that time is very fast.”*. At both institutions, ward physicians communicated more in‐depth with families than PICU providers, but less than nurses: *“our discussion was more in depth explaining the whole situation and why we were doing it and all those things”* (ward physician, St. Jude).

As nurses spent the most time at the bedside, the family became increasingly comfortable with them, leading to better communication: *“communication between us and the patient's family is very good because we are [always] with them, practically”* (nurse, UNOP). In addition, nurses at both institutions expressed providing additional education when physician communication was insufficient: *“First of all, there is the education staff, who go and give them a teaching topic and explain to them, but most of the time, or half the time they listen to a lot of words and see slides, but they don't fully understand. Then we have to explain again to the child, we have to explain again to the parents”* (nurse, UNOP).

#### 3.2.2 Family‐level barriers

At the level of the family, UNOP providers discussed language and educational barriers that impaired communication. These barriers to family communication were not expressed by participants at St. Jude.

Language barriers affected the ability of parents at UNOP to understand providers, limiting their involvement on the patient care team *“we have language barriers, so… Is difficult to tell them: ‘hi, I am going to move the patient because he has [a PEWS score] of 6’ they will not understand that type of terms” (*ward physician, UNOP), and the provider's ability to elicit and understand family concern: *“the part on [PEWS] where it asks about how the child is feeling, but if they don't speak Spanish [it is] more difficult to know how they feel*” (PICU provider, UNOP). Providers at UNOP also thought language barriers discouraged families from communicating: “*in cases where there has been a lack of interest [from the family] it is because of language barrier…here…there are many dialects*” (ward physician, UNOP). To overcome language barriers, staff at UNOP occasionally used informal interpreters: “*the security guards…many are indigenous and speak good Spanish…they [translate] to the respective language of the [family], how faithful is the flow of information, that's unknown*” (ward physician, UNOP). In other cases, providers resorted to interpreting concern based only on facial expressions: “*More than once I observed this, that when they ask the father about his opinion about the child, and they don't understand a word of Spanish, well we go to the limbic system, and if we see their little face a little pained then we can say they are preoccupied*” (PICU provider, UNOP).

Just as language discordance discouraged providers from spending time communicating with families, so did a perception that low medical literacy impeded understanding of medical communication: *“I believe [the family is] not very familiarized…we communicate; ‘look, the patient has tachycardia…’ [and] they hear us but don't pay much attention”* (ward physician, UNOP). Provider also perceived families felt shame or embarrassment due to their potential lack of understanding: *“we have problems because maybe the child is very sick, but the parent does not say anything because he feels embarrassed, ashamed, or cannot…express the ideas”* (ward physician, UNOP).

### 3.3 Impact of PEWS on family‐centered communication

Overall, providers perceived PEWS to improve the quality of family‐centered communication by requiring evaluation of family concern and eliciting family‐centered discussions and education that facilitated relationship‐building. This theme was described across all disciplines at both institutions, but it was particularly predominant at St. Jude where PEWS were more systematically explained to families. Table 4 describes examples of PEWS impacting provider‐family communication.

**TABLE 4:** Impact of PEWS on provider‐family communication

Scoring	*“Then you say to the family, you know we are worried about your child because we have this scoring system” (ward provider, St. Jude)* *“It has changed a lot, because you have more communication with the patient, maybe before [PEWS, it was on and off]” (nurse, UNOP)*
Relationship‐building	*“one good thing about communication with parents is if you have already seen a patient [in the ward], you have kind of built a relationship somewhat before they come up [to the PICU]” (PICU provider, St. Jude)* *“You get to communicate a lot with the family, they come to you to ask” (nurse, UNOP)*
Anticipatory guidance and education	*“when you have to call rapid response*…*we'll actually say, to*…*help alleviate, especially if it's a lower one*…*and the patient looks okay*…*’we are calling them in, you are about have all these people come in, this is why, this score is what's bringing them in’*…*and they do not get quite as worried” (nurse, St. Jude)* *“I usually explain it to the families*…*that we have this system that's in place that just allows the PICU team to know if there are changes” (PICU provider, St. Jude)* *“In the event of an alteration to the [PEWS] and there is a need to transfer him, the family member is always told, so it is for this reason that the patient will be transferred to intensive care” (nurse, UNOP)*
Negative impact	*“Sometimes I think the [PEWS] scores and having the fellows or whoever's on call overnight coming in in the middle of the night can be more distressing to parents.” (ward provider, St. Jude)* *“Because if you ask [the parent], he will always be [concerned]” (nurse, UNOP)* *“[Families ask], ‘Why are we now escalating care and they look the same?’” (ward provider, St. Jude)* *“[Families ask,] ‘If he is well, he looks good, why are they going to take him [to the PICU]’”? (nurse, UNOP)*

Abbreviations: PEWS, Pediatric Early Warning Systems; PICU, pediatric intensive care unit; St. Jude, St. Jude children's research hospital; UNOP, Unidad Nacional de Oncología Pediátrica.

#### 3.3.1 Scoring

The PEWS tool used at both institutions included a point for ‘family concern,’ which was perceived by providers to enhance communication by deliberately involving families in the care of their children and allowing providers and families to feel more comfortable communicating about the child's status: **“** *it feels like it does help parents ask more questions that they might not otherwise if we weren't doing these steps”* (ward physician, St. Jude). PEWS scoring provided a clear opportunity for providers to include families as part of the care team by eliciting their input and providing an opportunity to better understand their point of view: *“Not all family members show their concern in the same way because there are parents who are very quiet, sitting next to the child, but we do not know how they are unless we ask them questions”* (ward physician, UNOP).

#### 3.3.2 Relationship‐building

At St. Jude, PEWS also created opportunities for PICU providers to spend more time at the bedside, enabling relationship‐building with families: *“if you've been watching [a patient] and they get worse, they've already been introduced to the PICU team, so it might not be as stressful to them when they get transferred up to the unit…because we've had multiple discussions when it was a more relaxed environment”* (PICU provider, St. Jude). These pre‐transfer discussions elicited by abnormal PEWS allowed the family to be more comfortable when their child was transferred to the PICU. At UNOP, PICU providers also spoke to patients after assessments triggered by PEWS which require a physician consultation: *“The doctor who is in the service speaks to them, but after the assessment we talk to them as well. Always”* (PICU provider, UNOP).

#### 3.3.3 Anticipatory guidance and education

At. St. Jude, PEWS offered an opportunity for anticipatory guidance and family education. Providers used PEWS to reassure families that the PICU knew about their child's status: *“And I always leave the parents and families with…we're now aware of your child, they're on our radar…it helps destigmatize PICU”* (PICU provider, St. Jude). Before arrival of the PICU providers, nursing at St. Jude used PEWS as an opportunity to educate the family on PICU procedures: *“[the family is] still worried [because] you have everybody coming in, but it's a way to alleviate some of that…[by explaining that]…because it's reached this score, yes, he looks like he's doing fine…so, it's a way to open that communication”* (nurse, St. Jude). Providers at St. Jude also described how PEWS facilitated education of the family about their child's condition and treatment: *“on the side of the parents, you can…discuss ‐‐ with having that tool, you can sort of work through…this is why I'm going to try this intervention*” (ward physician, St. Jude).

At UNOP, there were less opportunities for anticipatory guidance through PEWS because the tool was not regularly explained to families; however, ward providers did explain PICU transfer: *“We don't explain the [PEWS] to the family because it's a complex matter and most of the population in this area is illiterate, we have language barriers, so… [it] is difficult to tell them…[and if] the nurse is checking him very often…they know that's not normal, in that case I explain to them [transfer procedures]”* (ward physician, UNOP).

#### 3.3.4 Negative impact

Providers less frequently mentioned potentially negative impacts of PEWS on interactions with families. At St. Jude, providers noted a potential for family alarm fatigue when families became less concerned over time due to multiple abnormal PEWS triggers without PICU transfer: *“if it's something that's getting triggered every four hours, the families kind of get sick of seeing us”* (PICU provider, St. Jude). At UNOP, providers mentioned that the family concern point was at times not accurate, because parents would be highly concerned regardless of the child's clinical status: *“Yes, there is a point of paternal concern, but it is more because he saw us running, not because he tells us, look, my son is different”* (ward physician, UNOP). Providers at both institutions mentioned that families were alarmed when a PEWS trigger resulted in PICU consultation even when the child was clinically stable (Table 4).

## 4 DISCUSSION

Providers across all disciplines at both institutions recognized families as a valuable part of the care team, particularly during events requiring escalation of care. Participants also identified several barriers to communication which could be addressed by targeted interventions, including standardizing communication responsibilities, increasing amount of time spent with families, obtaining designated professional interpreters and health educators, and using communication‐enhancing tools such as PEWS.

Results from St. Jude and UNOP demonstrate many similarities including their inclusion of the family as part of the care team, the provider‐level barrier of time spent interacting with the family, and the overall impacts of PEWS on family‐provider communication. Lack of time with the family has been previously described as a communication barrier in high‐resource settings.^7^ Our results build on this literature by demonstrating additional barriers in high‐resource settings and similar constraints in resource‐limited hospitals.

The two centers, however, differed in perceived family‐level barriers to communication. Language and educational barriers were reported only at UNOP, likely due to a lack of access to resources such as professional interpreters. Staff at UNOP tried to overcome language barriers by using family members or informal interpreters, however, this did not guarantee accurate and unbiased communication. The clinician perspectives of the language barrier are an important lens through which to understand interactions with families. Studying the clinician perspective gives us insight into the potential biases held while interacting with families and can lead us to find ways to lessen or remove those biases. Language, cultural, and educational barriers exist in settings of all resource‐levels,^3^ and this study further demonstrates the importance of ongoing research and interventions targeting these aspects of patient care.

Importantly, our findings demonstrate how PEWS helped overcome communication barriers by providing an easily conveyable score that facilitated family understanding, increased provider interaction with families, and enabled relationship‐building, anticipatory guidance, and family education. These factors also allowed increased involvement of families in the care of their children during clinical deterioration events. Family involvement is particularly important in pediatrics where children, who cannot fully express their own concern, rely on their families to advocate on their behalf. Our results demonstrate that PEWS may facilitate family involvement, supporting previous literature on the role of families in identifying deterioration^21^ and monitoring patients in settings with limited nursing staffing.^22^

By increasing provider‐family communication, PEWS was perceived to improve the quality of care in both hospitals. Although family concern is a validated component of PEWS,^9^, ^16^, ^18^ some PEWS tools do not include this domain.^23^ Our findings emphasize the importance of including family concern as part of PEWS scoring. This study also identified an opportunity to improve family‐centered communication at UNOP and standardize how providers educate families about PEWS,^24^ PEWS scoring, and response to deterioration. This can be accomplished using patient education materials^25^ and a standardized and adaptable approach to PEWS implementation and trainings.

Although this qualitative study allowed us to understand the nuanced nature of provider's experiences and explore how they perceive family‐communication in their settings, it has several limitations. We included only healthcare providers and thus our results lack the direct perspective of patients and families. The analysis of provider‐family communication presented is from the perspective of clinicians. Future work should include families to understand their perspective of the quality of communication with providers and the impact of PEWS. For work involving family and patient participants, interpreters, psychologists, and social workers should be closely involved to help obtain accurate and contextually appropriate data. The impact of PEWS on time spent by the care provider during hospital rounds can be further explored. This study was conducted in two pediatric oncology hospitals, potentially limiting the transferability of findings to adult populations or non‐oncology settings. Interviews were conducted in English and Spanish and translated to English for analysis, potentially modifying meaning of some statements; however, this was mitigated by review of translations prior to analysis by a bilingual member of the research team. Interview responses may also have been influenced by social desirability bias^26^; however, this was minimized by selecting interviewers who were outside the participants' clinical setting or reporting hierarchy.

In conclusion, PEWS improve the quality of communication between providers and families by increasing interaction, relationship‐building, and trust, as well as facilitating involvement of families in patient care. These findings further demonstrate the importance of PEWS to improve the quality of care in both high‐resource and resource‐limited settings.

## AUTHOR CONTRIBUTION

Conceptualization: SR Gillipelli, DE Graetz, A Agulnik. Data Curation: M Garza, M Rodriguez, DJ Soberanis, AM Aceituno, JS Gattuso. Formal Analysis: SR Gillipelli, DE Graetz, M Garza, G Ferrara, A Agulnik. Funding Acquisition: A Agulnik, C Rodriguez‐Galindo. Writing – Original Draft Preparation: SR Gillipelli, DE Graetz, A Agulnik. Writing – Reviewing and Editing: All authors.

## FUNDING INFORMATION

American Lebanese Syrian Associated Charities (ALSAC).

## CONFLICT OF INTEREST

The authors have no conflict of interests to disclose.

## Supporting information


Figure S1–S3
Table S1

## Data Availability

The data that support the findings of this study are available from the corresponding author, DG, upon reasonable request.
