# Supplementary material for: Pediatric Early Warning Systems (PEWS) improve provider‐family communication from the provider perspective in pediatric cancer patients experiencing clinical deterioration
Source: Cancer Med. 2022 Sep 21;12(3):3634–43. doi: 10.1002/cam4.5210 (PMC9939098; doi:10.1002/cam4.5210)
Supplement: Supplementary file 1 — Figure S1–S3 Table S1 [file CAM4-12-3634-s001.docx]

**Supporting Material**

**Pediatric Early Warning Systems (PEWS) Improve Provider-Family Communication in Pediatric Cancer Patients Experiencing Clinical Deterioration**

Srinithya R. Gillipelli, BA^1,2^, Erica C. Kaye, MD^3^, Marcela Garza, MD^2^, Gia Ferrara, MSGH^2^, Mario Rodriguez, MD^4^, Dora Judith Soberanis Vasquez, RN^5^, Alejandra Mendez Aceituno, MD^6^, Federico Antillón-Klussmann, MD, PhD^4,7^, Jami S. Gattuso, MSN, RN^8^, Belinda N. Mandrell, PhD, RN^8^, Justin N. Baker, MD^3^, Carlos Rodriguez-Galindo, MD^2^, Asya Agulnik, MD, MPH^2*^, Dylan E. Graetz, MD, MPH^2*^

*These authors contributed equally

^1^Baylor College of Medicine, Houston, TX, United States of America

^2^St. Jude Children's Research Hospital, Department of Global Pediatric Medicine, Memphis, TN, United States of America

^3^St. Jude Children's Research Hospital, Division of Quality of Life and Palliative Care, Memphis, TN, United States of America

^4^Unidad Nacional de Oncología Pediátrica, Department of Oncology, Guatemala City, Guatemala

^5^Unidad Nacional de Oncología Pediátrica, Department of Nursing, Guatemala City, Guatemala

^6^Unidad Nacional de Oncología Pediátrica, Department of Critical Care, Guatemala City, Guatemala

^7^Francisco Marroquin University School of Medicine, Guatemala City, Guatemala

^8^St. Jude Children's Research Hospital, Department of Nursing Research, Memphis, TN, United States of America

| Item | Page |
| --- | --- |
| Supporting Figure 1. PEWS (EVAT) Scoring Tool | 2 |
| Supporting Figure 2. PEWS (EVAT) Sample Algorithm | 3 |
| Supporting Figure 3. Interview Guide | 4 |
| Supporting Table 1. Code Definitions | 5 |

**Supporting Figure 1. PEWS (EVAT) Scoring Tool**

**
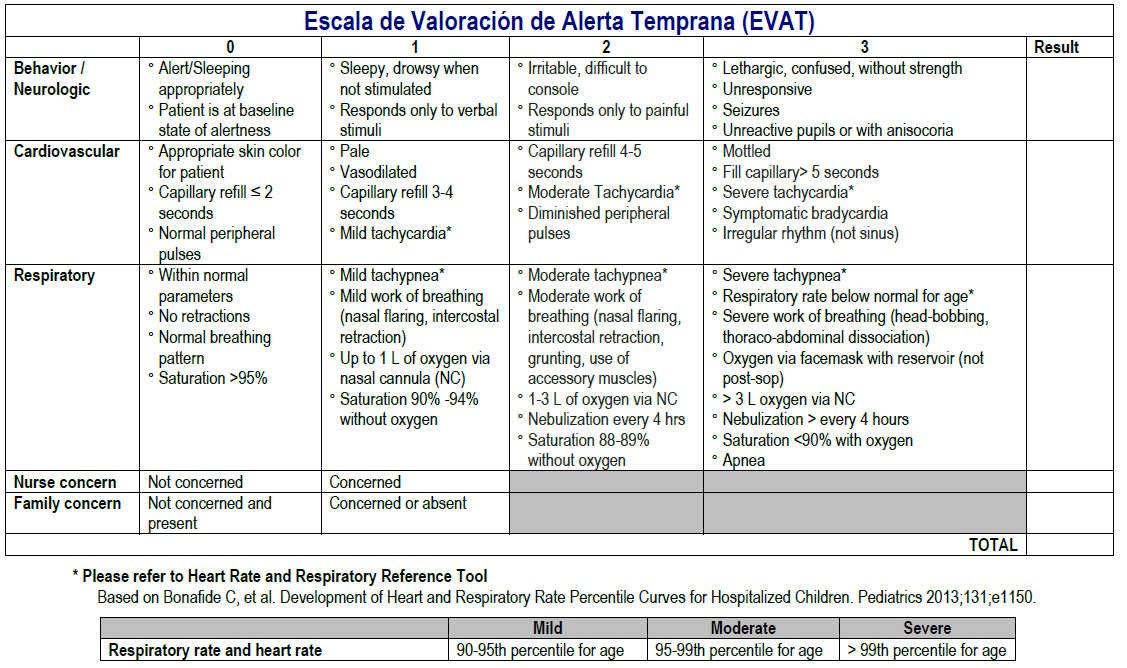
**

**Supporting Figure 2. PEWS (EVAT) Sample Algorithm
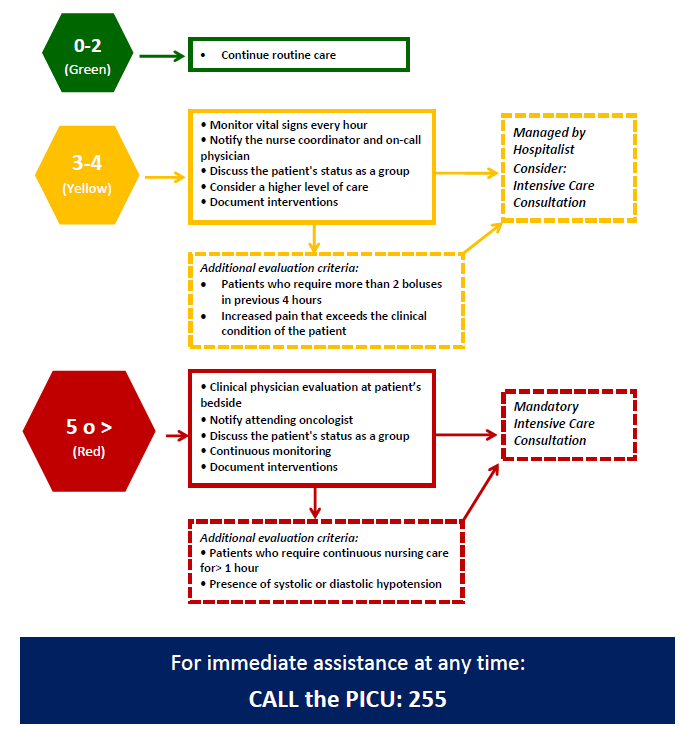
**

**Supporting Figure 3. Interview Script**

1. Tell me what you think about PEWS
   1. How does it work on your floor/unit?
   2. What is the role of PEWs in patient care?
   3. How does it (or doesn’t it) help you recognize patient deterioration?
   4. What do you like about it?
   5. What don’t you like about it?
   6. What would you change about it?
2. How does PEWs affect communication?
   1. How has it affected your work with...
      1. …nurses and nursing coordinators?
      2. ...residents, fellows and APPs?
      3. ....ICU team members?
      4. ...families?
   2. When you call a provider with a concern, do you include the PEWs score? (or: When a nurse calls you with a concern, do you ask for the PEWs score?)
      1. Why or why not?
      2. How do you decide when to score your concern under “staff concern”?
   3. When is PEWs helpful, when isn’t it?
3. What do you see as the barriers to communication around patient deterioration events?
   1. Can you think of a time when you thought about calling for help/escalating care and did not? (Can you think of a time when you feel care should have been escalated and was not?)?
   2. Why do you think this happened?
4. Were you at St. Jude prior to the implementation of PEWs? If yes....
   1. In what ways do you think it has affected patient safety?
   2. In what ways has it affected/changed communication?
   3. How do you think it has affected the safety culture at St. Jude?
5. Thank you for those thoughts. Now I’d like to talk to you about a specific event, is that okay? I’d like to talk to you about *event* on *day* can you tell me what happened?
   1. What was your shift/assignment like that day? How busy were you/was the team?
   2. What was the patient like? Tell me a bit about their history (e.g. prior deterioration events) and your history with them (did you take care of this patient regularly?)
   3. When did you first realize the patient was deteriorating? What happened next?
   4. Who was called first?
   5. Did the ICU team see the patient before, with, or after the floor providers?
   6. Who talked to the ICU team?
   7. Who made decisions regarding next steps? How were these decisions made? How were the decisions communicated? Were you comfortable with these decisions? Why or why not?
   8. How did the PEWs score help/hurt? How might have this event gone differently without SJAWS?
   9. In your opinion, did this case’s management go well? Is there anything you would have changed or wish had gone differently?
6. Is there anything else you would like to say about PEWs scores and their use here?

**Supporting Table 1. Code Definitions**

| **Category** | **Code** | **Definition** |
| --- | --- | --- |
| **Interdisciplinary Communication** | Family Communication | All communication directed to or received from the FAMILY regarding the patient’s care |
| **Qualitative Tool** | Parent concern | Any references to the point given on sJAWS/EVAT for “parent concern” |
